# Supplementary material for: CenSegNet: a generalist high-throughput deep learning framework for centrosome phenotyping at spatial and single-cell resolution in heterogeneous tissues
Source: Nat Commun. 2026 Jul 15;17:8713. doi: 10.1038/s41467-026-75393-y (PMC13490574; doi:10.1038/s41467-026-75393-y)
Supplement: Supplementary file 2 [file 41467_2026_75393_MOESM2_ESM.pdf]

File name: Supplementary Data 1. Table 1. Baseline characteristics of the cohort of patients with breast cancer.

File name: Supplementary Data 2. Light microscopy reporting table.
