# Supplementary material for: CenSegNet: a generalist high-throughput deep learning framework for centrosome phenotyping at spatial and single-cell resolution in heterogeneous tissues
Source: Nat Commun. 2026 Jul 15;17:8713. doi: 10.1038/s41467-026-75393-y (PMC13490574; doi:10.1038/s41467-026-75393-y)
Supplement: Supplementary file 3 [file 41467_2026_75393_MOESM5_ESM.pdf]

nature portfolio

Corresponding author(s): Stephen Beers, Xiaohao Cai, Salah Elias

Last updated by author(s): June 24 2026

Reporting Summary

Nature Portfolio wishes to improve the reproducibility of the work that we publish. This form provides structure for consistency and transparency in reporting. For further information on Nature Portfolio policies, see our Editorial Policies and the Editorial Policy Checklist.

Statistics

For all statistical analyses, confirm that the following items are present in the figure legend, table legend, main text, or Methods section.

n/a	Confirmed	
[ ]	[x]	The exact sample size (n) for each experimental group/condition, given as a discrete number and unit of measurement
[ ]	[x]	A statement on whether measurements were taken from distinct samples or whether the same sample was measured repeatedly
[ ]	[x]	The statistical test(s) used AND whether they are one- or two-sided<br>*Only common tests should be described solely by name; describe more complex techniques in the Methods section.*
[x]	[ ]	A description of all covariates tested
[ ]	[x]	A description of any assumptions or corrections, such as tests of normality and adjustment for multiple comparisons
[ ]	[x]	A full description of the statistical parameters including central tendency (e.g. means) or other basic estimates (e.g. regression coefficient) AND variation (e.g. standard deviation) or associated estimates of uncertainty (e.g. confidence intervals)
[ ]	[x]	For null hypothesis testing, the test statistic (e.g. F, t, r) with confidence intervals, effect sizes, degrees of freedom and P value noted<br>*Give P values as exact values whenever suitable.*
[x]	[ ]	For Bayesian analysis, information on the choice of priors and Markov chain Monte Carlo settings
[x]	[ ]	For hierarchical and complex designs, identification of the appropriate level for tests and full reporting of outcomes
[ ]	[x]	Estimates of effect sizes (e.g. Cohen's d, Pearson's r), indicating how they were calculated

Our web collection on statistics for biologists contains articles on many of the points above.

Software and code

Policy information about availability of computer code

Data collection	Inverted Leica STELLARIS 5 laser scanning confocal microscope (Leica Microsystems)<br>Zeiss Imager Z1 upright microscope equipped with an AxioCam MRc5 camera (Zeiss, Germany)
Data analysis	GraphPad Prism software (GraphPad, version 10)<br>Fiji (ImageJ 2.16.0/1.54p; Java 1.8.0_322, 64-bit)<br>Python (version 3.10)<br>PyTorch (version 2.1)<br>QuPath (version 0.5.1)<br>Cellpose (version 3.0)<br>CenSegNet (GitHub: https://github.com/SKELab/CenSegNet/; Zenodo: https://zenodo.org/records/17131573 (DOI: 10.5281/zenodo.17131572)

For manuscripts utilizing custom algorithms or software that are central to the research but not yet described in published literature, software must be made available to editors and reviewers. We strongly encourage code deposition in a community repository (e.g. GitHub). See the Nature Portfolio guidelines for submitting code & software for further information.

Data

Policy information about availability of data

All manuscripts must include a data availability statement. This statement should provide the following information, where applicable:

- Accession codes, unique identifiers, or web links for publicly available datasets
- A description of any restrictions on data availability
- For clinical datasets or third party data, please ensure that the statement adheres to our policy

Examples of immunohistochemistry and immunofluorescence image datasets generated in this study for benchmarking and testing CenSegNet within the demo version of the pipeline have been deposited in the Zenodo database under accession code 17131573 (https://doi.org/10.5281/zenodo.17131573) and are accessible via the project GitHub repository (https://github.com/SKELab/CenSegNet/). A representative set of immunohistochemistry (TMA cores) and immunofluorescence (confocal and lightning-based super-resolution) image datasets, together with the corresponding annotation files, have been deposited in the University of Southampton Institutional Repository accessible via (https://doi.org/10.5258/SOTON/D3960). All other data supporting the findings of this study are available within the article, its Supplementary Information and Supplementary Data files. The source data underlying all Figures and Supplementary Figures are provided as a Source Data file with this paper. A summary of the anonymised human breast samples used in this study, including baseline clinicopathological characteristics, is provided in Supplementary Data 1. Additional de-identified clinicopathological information for participants enrolled in the BeGIN study is available through a controlled-access process owing to participant privacy, consent and ethical governance requirements. Researchers seeking access should submit a completed Data Sharing request form describing a methodologically sound proposal, for the purpose of the approved research and in accordance with the initial patient consent. Requests will be reviewed by the study steering committee. Data will be shared once all parties have signed the relevant data-sharing documentation, covering the study steering committee conditions for sharing and, if required, an additional Data Sharing Agreement from the Sponsor. Requests should be addressed to R.I.Cutress@soton.ac.uk (see also Methods: Ethics and human breast tissues). All reagents generated in this study are available from the lead author upon request (s.k.elias@soton.ac.uk).

Research involving human participants, their data, or biological material

Policy information about studies with human participants or human data. See also policy information about sex, gender (identity/presentation), and sexual orientation and race, ethnicity and racism.

Reporting on sex and gender	Sex was considered in the study design, however breast cancer predominantly affects women, whereas male breast cancer accounts for approximately just under 1% of all breast cancer diagnoses. Male sex was not an exclusion criterion; all participants recruited into the BeGIN study were women. Sex was determined based on sex assigned at birth. Consequently, analyses according to sex or gender were not performed. Participant age was analysed and is reported in the baseline characteristics. Thus, our study complies with SAGER guidelines (https://www.nature.com/articles/s41467-022-30398-1).
Reporting on race, ethnicity, or other socially relevant groupings	N/A
Population characteristics	A summary of the anonymised human breast samples used in this study, including baseline clinicopathological characteristics (including age, genetic, T-stage, lymph node stage, histological tumour grade, histological tumour type, ER status, PR status, HER2 status, HER2 positivity, height, weight, waist, FFMI, FMI) is provided in Supplementary Data 1.
Recruitment	The study participants were a subgroup of women diagnosed with early breast cancer who were recruited to a single-centre prospective observational cohort study at University Hospitals Southampton, "Investigating Outcomes from Breast Cancer: Correlating Genetic, Immunological and Nutritional Predictors (BeGIN).
Ethics oversight	All procedures performed in studies involving human participants were in accordance with the ethical standards of the institutional and/or national research committee and with the 1964 Helsinki declaration and its later amendments or comparable ethical standards. All participants in BeGIN gave written informed consent and no financial compensation was provided for participation in the study. The research ethics committee approved the study (Research Ethics Committee (REC) - Cambridgeshire and Hertfordshire reference number: 14/EE/1297). This study follows best-practice reporting standards for tumour biomarker prognostic studies in line with the REMARK guidelines (https://www.nature.com/articles/ncponc0252). Women were eligible for the BeGIN study if they were aged >18 years and diagnosed with invasive breast cancer or DCIS at University Hospital Southampton after May 2015. Tissue blocks are stored in the University of Southampton Tissue Bank for the duration of the BeGIN study in compliance with the UK Human Tissue Act (HTA) and under HTA-compliant University governance regulating tissue storage, tracking, access, experimental use and disposal. De-identified tissues from participants within the BeGIN cohort are available to researchers upon request, subject to completion of a Material Transfer Agreement, appropriate ethical approval consistent with the original patient consent, and submission and approval of a methodologically sound research proposal. Requests will be reviewed by the study steering committee, and tissues will be shared following completion of all required material-sharing documentation. Anonymous data from the BeGIN study is available for researchers who submit a completed Data Sharing request form that describes a methodologically sound proposal consistent with the original patient consent, for the purpose of the approved proposal. Proposals will be reviewed by the study steering committee and, where approved, data will be shared once all parties have signed relevant data-sharing documentation, covering the study steering committee conditions for sharing and if required, an additional Data Sharing Agreement from the Sponsor. Requests should be addressed to R.I.Cutress@soton.ac.uk.

Note that full information on the approval of the study protocol must also be provided in the manuscript.

Field-specific reporting

Life sciences study design

All studies must disclose on these points even when the disclosure is negative.

Sample size

No formal a priori hypothesis-testing-based sample-size calculation was performed for development of the centrosome segmentation model or for downstream biological analyses based on model-derived measurements. This study used a prospective observational cohort comprising 127 consecutive breast cancer patients who met predefined inclusion criteria, including availability of tumour material, sufficient tissue quality, adequate staining and imaging quality, interpretable data, and availability of the required annotations and clinicopathological information. The cohort included the major breast cancer subtypes represented in the study population, including ER-positive tumours (n = 102, 80.3%), HER2-positive tumours (n = 18, 14.2%), and ER-negative PR-negative, and HER2-negative tumours (n = 7, 5.5%).

The patient sample was considered the independent biological unit. Multiple TMA cores, images, cells, or centrosomes derived from the same patient were used to improve sampling of intratumoural heterogeneity and technical robustness, but were not treated as independent biological replicates. This sample size was considered sufficient because it included all eligible patient samples available for the study and captured inter-patient variation in tumour morphology, staining intensity, centrosome abundance, tissue quality, and imaging characteristics.

For development and evaluation of the centrosome segmentation model, data partitioning and/or cross-validation were performed at the patient level to avoid information leakage between training and evaluation datasets. Model performance was assessed using patient-level segmentation and detection metrics, including Dice coefficient, IoU, F1 score, centrosome-detection sensitivity, with uncertainty estimated using 95% confidence intervals resampling.

For downstream biological analyses, all eligible patient samples were included, and analyses were performed using patient-level summaries of model-derived centrosome measurements. Effect sizes and 95% confidence intervals were reported where applicable to indicate the magnitude and uncertainty of observed associations. Statistical analyses accounted for the non-independence of multiple cores, images, cells, or centrosomes from the same patient by summarizing measurements at the patient level and/or using statistical approaches appropriate for nested data.

Experiments using mammary epithelial cells lines MCF10A, MCF10A-PLK4 and MCF10DCIS.com, and TUBO mouse tissues, were performed at least in 3 biological replicates, with multiple technical replicates per experiment. Sample size was determined on the basis of previous studies with similar experiments (Elias et al., 2014 Stem Cell reports; Elias et al., 2015 PLOS Biology; Fankhaenel et al., 2023 Nature Communications; Hosawi et al., 2024 Journal of Cell Science).

Sample sizes are reported in the figures and figure legends.

Data exclusions

Potential self-selection bias was minimised by the consecutive recruitment of eligible patients, rather than selective enrolment based on disease severity, image quality, or other clinical characteristics. In addition, the researchers involved in image analysis were blinded to the clinical characteristics of the patients, reducing the risk that clinical information could influence image processing or model assessment.

A potential source of selection bias was introduced during the image-processing stage used to train the CenSegNet model. Whole-slide images were cropped into patches, and patches with substantial artefact or poor image quality were manually excluded to maintain the integrity of the training dataset. However, we consider the likely impact of this bias to be limited. First, the excluded patches represented a relatively small proportion of the dataset (14.1%). Second, exclusion of severely artefactual or non-interpretable regions is a standard quality-control step for supervised model training, where poor-quality inputs may introduce label noise and impair model learning. Third, and most importantly, the trained model was subsequently evaluated on images with different tissue types and varying image quality. The model maintained good performance across these settings, suggesting that the exclusion of poor-quality training patches did not materially compromise generalisability.

Overall, although some degree of selection bias may have been introduced during training patch selection, this was unlikely to substantially affect the study conclusions. The use of consecutive patient recruitment, blinding to clinical characteristics, and validation across heterogeneous image types and qualities reduced the likelihood that self-selection or image-quality-related bias materially influenced the reported results.

Replication

1. Immunofluorescence experiments:
All immunofluorescence experiments in main figures and supplementary figures were performed with at least three biological replicates, and all replication attempts were successful. Experimental reproducibility was supported by multiple quality-control measures, and key findings were validated using complementary approaches.

2. Immunohistochemistry experiments:
We performed immunohistochemistry using tissue microarrays (TMAs) taken from surgical blocks from patients with breast cancer. A total of 911 sample cores were systematically sampled from three distinct, pathologically classified regions for each patient: tumour tissue (Tumour), tumour margin (Edge), and tumour-free tissue (Normal) from 127 patients. Each patient represented by three technical replicate cores from Tumour, Edge, and Normal regions, were procured for analysis. Each 0.6 mm in diameter and 5 µm in thickness were extracted from formalin-fixed, paraffin-embedded specimens and arrayed into recipient tissue microarray (TMA) blocks. This tri-regional, triplicate-core sampling strategy was designed to provide a comprehensive and robust representation of the tissue heterogeneity within and around the tumour microenvironment. In addition, biological replicates (n=127 patients) minimise random error.

Reproducibility was supported by the use of standardised experimental protocols, predefined quality-control criteria, and independent experimental repeats where applicable. Cell segmentation was performed using StarDist, an established segmentation method, and segmentation outputs were visually inspected for accuracy following analysis. Segmentation accuracy was further evaluated using the cell

membrane marker E-cadherin to confirm the assignment of centrosomes to their corresponding cells. Representative imaging experiments were independently repeated with similar results, as indicated in the relevant figure legends.

Assay failure was defined a priori as failure to meet protocol-specified quality-control criteria, including insufficient sample material, poor sample quality, failed internal controls, incomplete assay processing, or non-interpretable output. No assay-level failures requiring sample exclusion or repeat testing occurred in this study. All samples entering the assay workflow generated interpretable results and were retained for statistical analysis. Independent replication attempts, where performed, yielded consistent results.

Randomization

The primary goal of this study was to develop and validate CenSegNet, a deep learning model for centrosome and epithelial tissue segmentation in human breast cancer TMAs, mammary tissues from TUBO mice, and MCF10A cells.

For human tissue microarray (TMA) analyses, no participants were randomly assigned to treatment, control, or other experimental groups, as the study did not involve an interventional or prospective group allocation design. Instead, model training, validation, and testing were performed using annotated tissue images, and subsequent application to breast cancer TMAs was observational, using CenSegNet-derived segmentations and existing clinicopathological annotations. Random allocation was therefore not relevant to this component of the study. Where biological or clinical associations were assessed, relevant covariates were accounted for in downstream statistical analyses rather than controlled through experimental group allocation.

Similarly, no random allocation of TUBO mice into experimental groups was performed because the mouse samples were not used in a mouse-level intervention or treatment-group comparison. Mice were not separated into distinct experimental groups; rather, mouse-derived tissues were used as biological material for imaging and analysis under uniform experimental and processing conditions. Allocation at the organism level was therefore not relevant. Sample handling, staining, imaging, and analysis procedures were standardised across samples to minimise technical variability, rather than controlling covariates through group allocation.

For experiments using MCF10A mammary epithelial cell lines (MCF10A, MCF10A-PLK4, MCF10DCIS.com), two experimental frameworks were employed. For functional experiments (e.g., MCF10A and MCF10A-PLK4 control versus Centrinone-treated conditions), cells were randomly allocated to the relevant experimental groups prior to treatment, staining, imaging, or analysis, as appropriate for each experiment. In parallel, MCF10A, MCF10A-PLK4 and MCF10DCIS.com cells were also used for the development and validation of CenSegNet, where allocation into experimental groups was not relevant. Across all cell-based experiments, where multiple coverslips, wells, fields of view, or images were acquired, these were assigned and analysed using the same predefined criteria across experimental conditions to ensure consistency and minimise technical bias.

Blinding

Potential self-selection bias was minimised by the consecutive recruitment of eligible patients, rather than selective enrolment based on disease severity, image quality, or other clinical characteristics. Researchers were not blinded to group allocation during experiments and outcome assessment. Blinding was not possible as for each experiment the same researcher both generated and analysed the data.

Reporting for specific materials, systems and methods

We require information from authors about some types of materials, experimental systems and methods used in many studies. Here, indicate whether each material, system or method listed is relevant to your study. If you are not sure if a list item applies to your research, read the appropriate section before selecting a response.

Materials & experimental systems

n/a	Involved in the study
☐	☒ Antibodies
☐	☒ Eukaryotic cell lines
☒	☐ Palaeontology and archaeology
☐	☒ Animals and other organisms
☐	☒ Clinical data
☒	☐ Dual use research of concern
☒	☐ Plants

Methods

n/a	Involved in the study
☒	☐ ChIP-seq
☒	☐ Flow cytometry
☒	☐ MRI-based neuroimaging

Antibodies

Antibodies used

For immunofluorescence staining:
The following primary antibodies were used:
1. anti-GT335 (1:800; Adipogen, AG-20B-0020-C100),
2. anti-pericentrin (1:250; Abcam, ab4448),
3. anti-keratin 8/18 (K8/18; 1:300; Origene, BP5007),
4. anti-keratin 8/18 (K8/18; 1:50; Dako, IR094)
5. anti-E-cadherin (20 ug/ml, Invitrogen #13-1900).

The following secondary antibodies (Life Technologies) were used:
1. goat anti-mouse (#A-32723) conjugated to AlexaFluor488 at 5 ug/ml.
2. goat anti-rabbit (#A-11008) conjugated to AlexaFluor488 at 5 ug/ml.
3. goat anti-rabbit (#A-11037) conjugated to AlexaFluor594 at 5 ug/ml.

	4. goat anti-rat (#A-21094) conjugated to AlexaFluor 633 at 5 ug/ml.<br>5. goat anti-guineapig (#A-21450) conjugated to AlexaFluor 647 at 5 ug/ml.<br><br>For immunohistochemistry staining:<br>The primary antibodies anti-pericentrin (1:500; Abcam, ab4448) and anti-K8/18 (1:50; Dako, IR094) were used. Detection of the primary antibody was achieved using EnVision FLEX HRP and Rabbit Link (Dako) secondary reagents according to instructions of the manufacturer.
Validation	Primary antibodies used in this study have been validated using immunohistochemistry and immunofluorescence and tested for specificity by their respective suppliers and by previous peer-reviewed publications (e.g., (Elias et al., 2014 Stem Cell reports; Elias et al., 2015 PLOS Biology; Fankhaenel et al., 2023 Nature Communications; Hosawi et al., 2024 Journal of Cell Science; Patel et al., 2018 Nature Communications). In our experiments, we further validated the antibodies (in terms of cellular localisation) by immunohistochemistry and immunofluorescence. As detailed below, this validation information is provided on the suppliers' webpages for each antibody:<br>1. anti-GT335 (1:800; Adipogen, AG-20B-0020-C100), https://adipogen.com/ag-20b-0020-anti-polyglutamylation-modification-mab-gt335.html. This antibody was recommended by Professor Susana Godinho (The Barts Cancer Institute, QMUL, UK), a leading expert in centrosome biology, whose lab has also validated the antibody using immunofluorescence in mammary cells and tissues.<br>2. anti-pericentrin (1:250; Abcam, ab4448), https://www.abcam.com/en-us/products/primary-antibodies/pericentrin-antibody-centrosome-marker-ab4448.<br>3. anti-keratin 8/18 (K8/18; 1:300; Origene, BP5007), https://www.origene.com/catalog/antibodies/primary-antibodies/bp5007-cytokeratin-8-krt8-krt18-guinea-pig-polyclonal-antibody.<br>4. anti-keratin 8/18 (K8/18; 1:50; Dako, IR094), https://www.agilent.com/en/product/immunohistochemistry/antibodies-controls/primary-antibodies/cytokeratin-8-18-%28autostainer-link-48%29-76312?srsltid=AfmBOooVHOt4efaednXpl1BZ01NIvMbom1fk-NJUK-2hN_QBCuVgV8fP.<br>5. anti-E-cadherin (20 ug/ml; Invitrogen, 13-1900), https://www.thermofisher.com/antibody/product/E-cadherin-Antibody-clone-ECCD-2-Monoclonal/13-1900.

Eukaryotic cell lines

Policy information about cell lines and Sex and Gender in Research

Cell line source(s)	MCF-10A cell line was purchased from (ATCC® #CRL-10317).<br>MCF10A-PLK4 and MCF10DCIS.com cell lines were provided by Prof. Susana A. Godinho (The Barts Cancer Institute, QMUL, UK), originally generated as described: https://www.nature.com/articles/nature13277.
Authentication	MCF-10A cells were verified by checking their morphology and the expression of epithelial markers such as E-cadherin, keratin 8 and keratin 14. MCF10A-PLK4 cells were verified based on morphological features and centrosome amplification status before and after doxycycline treatment, but have not been authenticated at the molecular level.<br><br>MCF10DCIS.com cell line was verified based on morphological features and altered polarity as compared to MCF10A and authenticated by RNA-seq.
Mycoplasma contamination	All cell lines were tested negative for mycoplasma contamination.
Commonly misidentified lines (See ICLAC register)	No commonly misidentified cell lines were used in this study.

Animals and other research organisms

Policy information about studies involving animals; ARRIVE guidelines recommended for reporting animal research, and Sex and Gender in Research

Laboratory animals	BALB/c HER2/neu transgenic mice (referred to as BALB-NeuT) carrying the transforming rat Her2/neu oncogene under control of a MMTV-LTR were used. Mice were housed in a specific pathogen-free (SPF) facility under controlled environmental conditions, including regulated temperature and humidity, with a 12-hour light/dark cycle. Animals had ad libitum access to standard chow and water.
Wild animals	No wild animals were used in this study.
Reporting on sex	Given that breast cancer predominantly affects females, all experimental procedures use female mice and were carried out under UK Home Office Establishment Licence 70/2906 XDA2EEF13, Project Licence P81E129B7 and Personal Licence 1918C6062 and approved by the University of Southampton Local Ethics Committee and registered with the Ethics and Research Governance Online II (ERGO II; ID: 64937). All animal work was conducted in accordance with UK Home Office regulations, adhering to the principles of the 3Rs (Replacement, Reduction, Refinement) and the ARRIVE (Animal Research: Reporting of In Vivo Experiments) guidelines to minimise animal suffering throughout the study.
Field-collected samples	No field collected samples were used in this study.
Ethics oversight	This study utilised archived fresh-frozen in Optimal Cutting Temperature (OCT) compound tumour samples that were originally generated as follows. TUBO cells (2 x 105) established from a spontaneous lobular carcinoma in BALB/c HER2/neu transgenic mice100 were subcutaneously injected into BALB/c (n = 50, 8-12-week-old females) mice. Given that breast cancer predominantly affects females, all experimental procedures use female mice and were carried out under UK Home Office Establishment Licence 70/2906 XDA2EEF13, Project Licence P81E129B7 and Personal Licence 1918C6062 and approved by the University of Southampton

Local Ethics Committee and registered with the Ethics and Research Governance Online II (ERGO II; ID: 64937). All animal work was conducted in accordance with UK Home Office regulations, adhering to the principles of the 3Rs (Replacement, Reduction, Refinement) and the ARRIVE (Animal Research: Reporting of In Vivo Experiments) guidelines to minimise animal suffering throughout the study. The maximum tumour size permitted under these approvals was 4000 mm3, equivalent to 400 mm2. No tumour bearing animals exceeded these limits.

Note that full information on the approval of the study protocol must also be provided in the manuscript.

Clinical data

Policy information about clinical studies

All manuscripts should comply with the ICMJE guidelines for publication of clinical research and a completed CONSORT checklist must be included with all submissions.

Clinical trial registration

The study was approved by the Research Ethics Committee (REC, Cambridgeshire and Hertfordshire reference number: 14/EE/1297).

Study protocol

This study was limited to quantifying centrosome abnormalities based on centrosomal protein expression in existing human tissue and cell samples; no clinical trial protocol was required.

Data collection

Centrosome abnormality status was quantified from immunofluorescence from mammary epithelial cells and BALB/c HER2/neu transgenic mice, and immunohistochemistry images from human breast tissue TMAs using a newly developed computational model (CenSegNet, https://github.com/SKELab/CenSegNet); Performance of CenSegNet was validated across multiple image datasets and analyzed using Python (version 3.10).

Outcomes

We present CenSegNet (Centrosome Segmentation Network, https://github.com/SKELAB/CenSegNet), a modular deep learning framework for high-resolution, context-aware segmentation of centrosomes and epithelial architecture across diverse tissue types. This platform enables scalable, generalizable, spatially resolved centrosome phenotyping in intact tissues, facilitating systematic analysis of centrosome biology and its dysregulation in cancer and other epithelial diseases. Structural centrosome abnormalities (Stru CA) and numerical centrosome abnormalities (Num CA), defined by centrosome size and number per cell, respectively. These CA subtypes were analysed for spatial distribution, age-dependent dynamics, and associations with histological tumour grade, hormone receptor status, genomic alterations, and nodal involvement. We show that Stru CA are associated with overall survival, whereas discordant CA profiles at tumour margins correlate with local tumour aggressiveness and stromal remodelling, highlighting their potential clinical relevance.

This study adheres to REMARK guidelines and Nature Portfolio reporting standards for tumour-marker prognostic studies (https://www.nature.com/articles/ncponc0252). We provided transparent information on the study population, specimen handling, marker assessment, endpoint definitions, follow-up, statistical analysis, and interpretation of the findings. In line with REMARK recommendations, the marker threshold was defined using the present dataset and considered alongside published evidence before classifying patients for outcome analysis. this data-driven approach is described transparently and interpreted with appropriate caution. The need for independent validation to confirm robustness and generalisability is explicitly acknowledged.

Plants

Seed stocks

N/A

Novel plant genotypes

N/A

Authentication

N/A
