# Supplementary material for: CenSegNet: a generalist high-throughput deep learning framework for centrosome phenotyping at spatial and single-cell resolution in heterogeneous tissues
Source: Nat Commun. 2026 Jul 15;17:8713. doi: 10.1038/s41467-026-75393-y (PMC13490574; doi:10.1038/s41467-026-75393-y)
Supplement: Supplementary file 4 [file 41467_2026_75393_MOESM6_ESM.pdf]

nature portfolio

# Peer Review File

# CenSegNet: a generalist high-throughput deep learning framework for centrosome phenotyping at spatial and single-cell resolution in heterogeneous tissues

Corresponding Author: Dr Salah Elias

**This file contains all reviewer reports in order by version, followed by all author rebuttals in order by version.**

Version 0:

Reviewer comments:

Reviewer #1

(Remarks to the Author)
The work by Cheng et al aims to fill a significant gap in cancer cell biology and diagnostics, aiming to provide an advanced software tool that will allow to predict centrosome aberrations in tumor tissue, based on AI_supported analysis of images of H&E sections additionally stained with antibodies recognizing pericentrin, a key component of the pericentriolar material (PCM) surrounding centrosomes. The software has also been trained to quantify centrosomes identified in immunofluorescence analysis and seems to outperform currently available software.

A reliable computational tool for centrosome quantification in tissues and cell lines would be very valuable for different fields, going beyond tumor diagnostics. The computational centrosome annotations based on pericentrin staining in IHC or IF seem reliable. However, there are major concerns regarding the data interpretation and conclusions draw that do not find support by the provided image analyses

The authors state that CA refers to centrosome amplification, so it is unclear what exactly “structural CA” means. What they describe as structural CA, in contrast to numerical CA, is at most structural aberrations but not necessarily coinciding with amplification of centrosomes. This needs clarification.

They claim that differences in centrosome size, i.e. pericentrin foci size, correspond to differences in centrosome structure is not really convincing. The microscopy images showing centrosome staining in paraffin sections, cryosections, and cell lines were acquired using insufficient magnifications to show centriole over-elongation or fragmentation (IHC 20x, immunofluorescence 40x objectives). To visualize true structural aberrations, techniques such as electron microscopy, super-resolution microscopy, or expansion microscopy are needed for training and analysis. To address that shortcoming, detailed imaging and measurements of centrioles and centrosomes using advanced microscopy techniques with sufficient resolution are needed to support the interpretation on structural aberrations in different tumor regions and across patient ages.

The authors exclusively use pericentrin staining to visualize centrosomes in tissues. Pericentrin is a component of the PCM and hence the antibody does not stain centrioles directly. Importantly, the PCM also varies in size between cell cycle phases. Hence, differences in foci size could also indicate different cell cycle states and not differences in centrosome counts. An additional centriolar marker should be added/compared to confirm their results. Moreover, acentriolar MTOCs can contain pericentrin and may hence score as false positives.

An alternative interpretation of the data not considered here is that larger foci represent clusters of centrosomes. This is supported by the observation described in line 208 (Suppl Fig. 3b): “At the single-cell level, increasing centrosome number per cell was associated with a reduction in the size of individual centrosomes (Supplementary Fig. 3b), which become smaller, more uniform in cells containing >4 centrosomes—implying a compensatory constraint on total centrosome volume.”
While the authors compare a series of clinical parameters, including hormone status or HER2, and correlate them with pericentrin-positive dots, simple correlations with patient overall or treatment-free survival, or treatment response in general, are missed. This seems possible to include and of significant value, assuming that the authors would like to use this software as a predictive tool.

Sometimes the number of centrosomes per cell estimated appears extremely high (8=16 centrioles), leaving the possibility that DAB substrate aggregates may score as “centrosomes”, in particular as signal amplification and enzymatic detection has been routinely used. This needs to be excluded as a confounding factor. Background signals may be better estimated using paraffin embedded cells pretreated with PLK4 inhibitors, to deplete centrioles. On the other hand, rather than having PLK4 overexpression to amplify centrioles in an uncontrolled manner, induction of cytokinesis defects would allow linear scaling of centrosome numbers and may be useful to create internal standards for training.

In summary, the CenSegNet seems to be a valuable tool for quantifying centrosomes in tissues. However, since structural aberrations are impossible to see with the microscopy techniques used here, we doubt the interpretation of the respective data (Figs 3-5). The use of antibodies recognizing actual centriolar proteins, such as CP110 or alike, are recommended to be included in addition to or to replace pericentrin.

(Remarks on code availability)

Reviewer #2

(Remarks to the Author)
I co-reviewed this manuscript with one of the reviewers who provided the listed reports. This is part of the Nature Communications initiative to facilitate training in peer review and to provide appropriate recognition for Early Career Researchers who co-review manuscripts.

(Remarks on code availability)

Reviewer #3

(Remarks to the Author)
• Include functional assays (e.g., perturbation of PLK4, AURKA, or PCM components) to validate the inferred mechanistic uncoupling of numerical and structural CA.
• Alternatively, clarify more explicitly in the Discussion that conclusions are observational and hypothesis-generating.
• Provide quantitative CA analyses or clinical correlations for at least one additional epithelial cancer type.
• Alternatively, reframe claims of generalisability more conservatively.
• Include inter-observer agreement metrics (e.g., IoU, Cohen’s κ) on a subset of data.
• Clarify whether annotations were reviewed or validated by multiple experts.
• Provide sensitivity analyses showing how varying this threshold affects conclusions.
• Justify modality-specific calibration or confirm robustness across IF and IHC datasets.
• Include multivariate analyses (e.g., Cox regression or logistic regression) controlling for standard prognostic factors.
• Clarify whether CA provides independent predictive value.
• Clarify inference-time requirements for typical users.
• Provide benchmarks on lower-end GPUs or CPU-only inference where possible.
•

(Remarks on code availability)
The README file is provided and source code contains all the functionalities incorporated.
It provide the demo video for GUI execution.

Version 1:

Reviewer comments:

Reviewer #1

(Remarks to the Author)
We want to thank the authors for the time and effort taken to address our concerns. We believe that this tool will be of merit to define centrosome alterations in cancer as a marker of tumor progression or therapy responses at large scale, hopefully settling an ongoing debate on the role of centrosomes in cancer formation and evolution.

(Remarks on code availability)

Reviewer #3

(Remarks to the Author)
The author devoted to this work and gave response for each comment.

(Remarks on code availability)

**Open Access** This Peer Review File is licensed under a Creative Commons Attribution 4.0 International License, which permits use, sharing, adaptation, distribution and reproduction in any medium or format, as long as you give appropriate credit to the original author(s) and the source, provide a link to the Creative Commons license, and indicate if changes were made.
In cases where reviewers are anonymous, credit should be given to 'Anonymous Referee' and the source.
The images or other third party material in this Peer Review File are included in the article's Creative Commons license, unless indicated otherwise in a credit line to the material. If material is not included in the article's Creative Commons license and your intended use is not permitted by statutory regulation or exceeds the permitted use, you will need to obtain permission directly from the copyright holder.
To view a copy of this license, visit https://creativecommons.org/licenses/by/4.0/

**POINT-BY-POINT RESPONSE TO THE REVIEWERS' COMMENTS**

We are grateful for the Reviewers' thoughtful comments and constructive suggestions, which have substantially contributed to improving the revised manuscript. We have carefully addressed all the Reviewers' concerns in the point-by-point responses below. In doing so, we have performed additional experiments and analyses, revised existing figures, added new figures, and modified the text to clarify data interpretation and to ensure that our conclusions are fully supported by the results. All textual changes in the revised manuscript are marked in Blue.

**Reviewer #2 (Remarks to the Author):**

I co-reviewed this manuscript with one of the reviewers who provided the listed reports. This is part of the Nature Communications initiative to facilitate training in peer review and to provide appropriate recognition for Early Career Researchers who co-review manuscripts.

- We thank Reviewer #2 for the time and care invested in evaluating our manuscript. We also acknowledge and appreciate the *Nature Communications* initiative supporting co-review and training in peer review for Early Career Researchers. Please find our detailed, point-by-point responses to the Reviewer's comments below.

**Reviewer #1 (Remarks to the Author)**

The work by Cheng et al aims to fill a significant gap in cancer cell biology and diagnostics, aiming to provide an advanced software tool that will allow to predict centrosome aberrations in tumor tissue, based on AI_supported analysis of images of H&E sections additionally stained with antibodies recognizing pericentrin, a key component of the pericentriolar material (PCM) surrounding centrosomes. The software has also been trained to quantify centrosomes identified in immunofluorescence analysis and seems to outperform currently available software.

A reliable computational tool for centrosome quantification in tissues and cell lines would be very valuable for different fields, going beyond tumor diagnostics. The computational centrosome annotations based on pericentrin staining in IHC or IF seem reliable. However, there are major concerns regarding the data interpretation and conclusions draw that do not find support by the provided image analyses

1) The authors state that CA refers to centrosome amplification, so it is unclear what exactly "structural CA" means. What they describe as structural CA, in contrast to numerical CA, is at most structural aberrations but not necessarily coinciding with amplification of centrosomes. This needs clarification.

- As Reviewer #1 rightly pointed out, centrosome amplification is often used to describe amplification/duplication of centrosomes, and that structural abnormalities do not necessarily arise from amplification. As the Reviewer notes, we had already clarified this distinction in the initial submission (see revised Introduction, lines 68–72), where we described the mechanisms driving structural centrosome abnormalities—such as aberrant pericentriolar material (PCM) accumulation and centriolar architectural abnormalities—supported by several published studies. In those lines, we also explained that certain architectural defects, including centriole over-elongation and fragmentation, can destabilise centriole integrity and promote overduplication, thus suggesting a mechanistic link between structural and numerical centrosome abnormalities.

Nonetheless, we recognise that the distinction may not have been sufficiently emphasised, and we have therefore clarified this terminology more explicitly in the revised manuscript.

Specifically, we now use the abbreviation CA to refer to **centrosome abnormalities/aberrations**, which can be either numerical (numerical CA) or structural (structural CA), in closer alignment with published terminology (Godinho and Pellman 2014 PMID: 25047621; Godinho and Basto 2025 PMID: 40274495; LoMastro and Holland 2019 PMID: 31063752). Within this framework, numerical CA refers to amplification/duplication of centrosomes, whereas structural CA refers to PCM expansion detected by pericentrin labelling, reflecting morphological features consistent with centrosome architectural defects. For clarity, this terminology is used consistently throughout our point-by-point response to the Reviewers. In addition, consistent with the Reviewer's observation—and as addressed further under point 4—we now explicitly consider putative centrosome clustering (see response to Reviewer #1 point 4) and acknowledge that enlarged pericentrin-positive structures may also arise from clustering of smaller centrosomes. This clarification is now reflected in the revised text (revised Introduction line 69).

Importantly, as described in the initial Results section (revised lines 221–223), our definition of structural CA is based on a previously established pericentrin-based size criterion validated in human breast cancer tissues, in which such enlarged structures were absent in normal tissue (Patel *et al.* 2018 PMID: 29535384). We have now highlighted more clearly in the revised manuscript the rationale for the mechanistic distinction between numerical and structural CA adopted in our study (revised Results, lines 250–254), ensuring that the quantitative framework underpinning our analyses is fully transparent. This approach is further strengthened by additional orthogonal validation of CenSegNet across both chromogenic immunohistochemistry (IHC) and immunofluorescence (IF) imaging modalities (see response to Reviewer #1, point 3), together with explicit sensitivity analyses and robust, modality-specific image-processing and calibration (see responses to Reviewer #3, points 7 and 8), enabling high-performance, high-precision segmentation of centrosomes and quantitative assessment of their number and size.

2) They claim that differences in centrosome size, i.e. pericentrin foci size, correspond to differences in centrosome structure is not really convincing. The microscopy images showing centrosome staining in paraffin sections, cryosections, and cell lines were acquired using insufficient magnifications to show centriole over-elongation or fragmentation (IHC 20x, immunofluorescence 40x objectives). To visualize true structural aberrations, techniques such as electron microscopy, super-resolution microscopy, or expansion microscopy are needed for training and analysis. To address that shortcoming, detailed imaging and measurements of centrioles and centrosomes using advanced microscopy techniques with sufficient resolution are needed to support the interpretation on structural aberrations in different tumor regions and across patient ages.

- Building on the clarification provided in response to point 1 regarding our definition of structural CA, we fully agree with Reviewer #1 that pericentrin-defined foci size alone cannot resolve centriole-level ultrastructural features, such as centriole over-elongation or fragmentation. We also agree that definitive assessment of such ultrastructural architecture would require electron microscopy, super-resolution microscopy, or expansion microscopy. As our study is grounded in clinically accessible imaging modalities, namely paraffin-embedded (FPPE) chromogenic IHC and standard IF, our aim is not to infer centriole ultrastructure per se, but rather to quantify—at high throughput—recognised PCM-based phenotypes that have been validated as structural centrosome aberrations in large imaging datasets derived from complex and heterogeneous tumour tissues. This distinction is now made explicit in the revised manuscript (revised Discussion, lines 547-563).

To address the Reviewer's specific concern regarding image resolution, we clarify in the revised Methods (lines 869–877) that our imaging approach of TMA core IHC is designed to generate up to 8000 × 8000 high-resolution images capable of supporting single-cell–resolution, high-magnification insets, with consistent illumination and contrast across samples.

As shown throughout the Results section of the initial submission (e.g. **Supplementary Fig. 3; Fig. 5** now revised **Supplementary Fig. 4; Fig. 6**), these imaging conditions allow us to distinguish numerical CA from enlarged pericentrin-positive structures with a level of detail comparable to confocal microscopy. To illustrate this more explicitly, we now include an additional representative IHC TMA core with multiple corresponding high-magnification insets in the revised manuscript (revised **Supplementary Figure 18a**). In parallel, we broadened validation across imaging platforms by adding quantitative analyses demonstrating CenSegNet's consistent performance across IHC, confocal, and Lightning-based computational super-resolution imaging using the Leica STELLARIS platform imaging (revised **Supplementary Fig. 1e**; new **Fig. 3**), with corresponding revisions to the text (Results, lines 135–143 and 224–246; Methods, lines 883-885). These data strengthen our central conclusion that CenSegNet performs consistently across both lower- and higher-resolution imaging contexts, which is essential for its intended versatility and generalisability. We also wish to direct the Reviewer to our response to a related point raised by Reviewer #3 (point 8), where we describe our modality-specific calibration strategy for IHC and IF imaging. This approach ensures that the model receives appropriately normalised inputs for each imaging modality, thereby improving generalisability while minimising modality-driven bias.

Crucially, while electron microscopy, expansion microscopy, and super-resolution imaging provide unmatched ultrastructural centriolar detail, these approaches are inherently low-throughput, technically challenging, making their routine application at scale for cohort-level analysis of hundreds of patient-derived TMA FFPE specimens remains unrealistic. We have therefore clarified in the revised Discussion (lines 547–563) that CenSegNet is deliberately optimised for high-throughput, clinically applicable imaging modalities, while explicitly acknowledging that centriole ultrastructural features cannot be resolved using routine IHC or standard IF and will require targeted follow-up studies using higher-resolution approaches. These clarifications align our conclusions with the intrinsic resolution limits of the imaging modalities employed, while preserving the translational and cohort-scale focus of the study.

3) The authors exclusively use pericentrin staining to visualize centrosomes in tissues. Pericentrin is a component of the PCM and hence the antibody does not stain centrioles directly. Importantly, the PCM also varies in size between cell cycle phases. Hence, differences in foci size could also indicate different cell cycle states and not differences in centrosome counts. An additional centriolar marker should be added/compared to confirm their results. Moreover, acentriolar MTOCs can contain pericentrin and may hence score as false positives.

- Reviewer #1 rightly points out that pericentrin labels the PCM rather than centrioles directly, that PCM size is dynamically regulated across the cell cycle, and that acentriolar MTOCs can, in some circumstances, accumulate pericentrin and therefore represent potential false positives. We have now revised the manuscript to further clarify these aspects of centrosome biology in the revised Introduction (line 50) and Results (revised lines 143-144).

We wish to emphasise that our choice of pericentrin for FFPE tissue sections was driven by its robustness and reproducibility in paraffin-embedded clinical material. This was a critical technical consideration for the development and implementation of CenSegNet as a robust, automated, high-throughput framework for centrosome phenotyping in complex epithelial environments. Importantly, as already discussed in the Introduction of the initial submission, most existing centrosome-quantification tools are tailored to IF imaging and are not compatible with standard chromogenic IHC workflows used in clinical pathology, which substantially limits their diagnostic and translational applicability. Our approach directly addresses this gap and is therefore intentionally aligned with widely adopted IHC pipelines to maximise practical applicability at scale, while explicitly acknowledging the specificity limitations inherent to a PCM-based marker (see revised Discussion, lines 547–563).

To address the Reviewer's request for centriolar-level quantitative validation, we pursued two complementary strategies. First, we attempted GT335 staining directly in FFPE tissue sections during the initial submission; however, despite extensive optimisation, GT335 did not yield reliable or specific signal in paraffin-embedded material. We wish to emphasise that—as also discussed in response to point 6 below—throughout this study, we sought expert advice from Professor Susana Godinho (Queen Mary University of London), a leading expert in centrosome biology, who also confirmed well-recognised challenges in detecting centriolar epitopes under FFPE chromogenic IHC conditions. Moreover, to our knowledge, there are no published studies showing reliable centriole visualisation by chromogenic IHC in FFPE human tissues. A few studies have used immunofluorescence across several human FFPE tissues to visualise centriolar markers such as CEP135 and CEP170, while recognising the inherent low-throughput nature of the methodologies as a key limitation (Wang *et al.* 2019 PMID: 30699045; Denu *et al.* 2019 PMID: 29330283). To overcome this limitation inherent to paraffin-embedded tissue, we therefore performed orthogonal validation in alternative mammary cell models in which centriole detection by immunofluorescence is robust. As already included in the initial submission, we performed pericentrin co-labelling with the well-established centriolar marker GT335 in both mouse mammary tissues and human mammary epithelial cells, including MCF10A and doxycycline-induced MCF10A-PLK4 cells overexpressing PLK4 (**Supplementary Fig. 1**). We have now revised the manuscript to further clarify these co-labelling analyses and to highlight the accompanying quantitative correlation data demonstrating strong concordance between pericentrin-labelled PCM and GT335-positive centrioles (revised **Supplementary Fig. 1**). Crucially, we now include super-resolution and confocal, z-stacked immunofluorescence imaging in MCF10DCIS breast cancer cells, which again demonstrates consistent pericentrin–GT335 colocalisation, supported by quantitative correlation analyses, in a tumour context that recapitulates the centrosomal defects observed in our TMAs (revised **Supplementary Fig. 1e, f**). Together, these complementary analyses, combined with robust annotation (see new **Supplementary Fig. 2**) and image calibration strategies—now further clarified in the revised manuscript (Results, lines 129-156; Methods, lines 801 and 888-908 and 939-969)—provide additional confidence that CenSegNet is quantifying bona fide centrosomal structures.

4) An alternative interpretation of the data not considered here is that larger foci represent clusters of centrosomes. This is supported by the observation described in line 208 (Suppl Fig. 3b): "At the single-cell level, increasing centrosome number per cell was associated with a reduction in the size of individual centrosomes (Supplementary Fig. 3b), which become smaller, more uniform in cells containing >4 centrosomes—implying a compensatory constraint on total centrosome volume."

- We agree with Reviewer #1 that, particularly in tumour tissues, an apparently large pericentrin-positive focus could arise from several underlying scenarios, including (i) an enlarged or irregular PCM structure, (ii) closely apposed centrosomes, or (iii) true clusters of centrosomes. While we have noted this possibility in the initial submission, we have now clarified this point further in the revised manuscript (Introduction, lines 69).

To address the Reviewer's comment, we performed a morphometric comparison between normal-sized and enlarged centrosomal foci in our dataset. If enlarged structures primarily reflected unresolved centrosome clusters, we would expect them to exhibit reduced roundness, lower compactness, or greater morphological irregularity compared with single centrosomes (Zwicker *et al.* 2014 PMID： 24979791; Woodruff *et al.* 2017 PMID: 28575670). Instead, our quantitative analyses show that circularity and solidity were largely maintained across the range of centrosome sizes. Circularity and solidity values did not correlate significantly with size, and enlarged centrosomes did not differ significantly from smaller centrosomes in these parameters (new **Supplementary Fig. 6**). These observations indicate that most enlarged centrosomal foci observed in our tissue samples retain the round, compact morphology expected of single centrosomes. While we cannot entirely rule out that a minority of large foci

may result from centrosome clustering, our data suggest that clustering is unlikely to be the predominant explanation for the enlarged structures observed. We have now clarified this point in the revised manuscript (Results, lines 303–316; Discussion, lines 593-599).

5) While the authors compare a series of clinical parameters, including hormone status or HER2, and correlate them with pericentrin-positive dots, simple correlations with patient overall or treatment-free survival, or treatment response in general, are missed. This seems possible to include and of significant value, assuming that the authors would like to use this software as a predictive tool.

- We thank Reviewer #1 for this important and constructive suggestion. We have now performed additional analyses examining the relationship between centrosome aberration (CA) status and survival outcomes, including overall survival (OS) and recurrence-free survival (RFS). We assessed both numerical and structural CA within the tumour core and the tumour–normal edge region. In the edge region, neither numerical CA nor structural CA showed a statistically significant association with OS or RFS in the current cohort (new **Supplementary Fig. 12a–d**). Similarly, most CA measures in the tumour compartment did not show significant associations with OS or RFS (new **Supplementary Fig. 12e, g, h**).

Notably, however, lower levels of structural CA in the tumour core were significantly associated with more favourable OS (new **Supplementary Fig. 12f**). Beyond this statistically significant association, we observed several additional consistent patterns. Patients with lower numerical CA together with higher structural CA in the tumour core exhibited comparatively poorer OS trends (new **Supplementary Fig. 12e**), although this relationship did not reach statistical significance. These survival patterns corroborate our clinicopathological analyses from the initial submission, in which patients classified as high numerical CA / low structural CA exhibited the most favourable profiles, including the smallest tumour sizes (**Fig. 5i**). Although some tumours in this group showed higher absolute node counts, their pathological nodal stage remained N0 (**Fig. 5m**), supporting a less aggressive overall phenotype. In contrast, patients with low numerical CA / high structural CA—who showed less favourable OS outcomes—exhibited larger tumours (**Fig. 5j**) and were enriched for a first- or second-degree family history of breast cancer (**Fig. 5o**).

Accordingly, while the current cohort remains limited in size and number of outcome events, the identification of a statistically significant association between tumour-core structural CA and OS, together with consistent clinicopathological correlations, suggests that spatially resolved CA phenotypes—particularly structural CA—capture biologically meaningful differences in tumour behaviour. We have incorporated these updated survival analyses and interpretations in the revised Results (lines 397–437) and Discussion (lines 525–532 and 704–735), and explicitly state that CenSegNet-enabled validation in larger, clinically well-annotated cohorts with extended follow-up will be required to establish the independent prognostic or predictive value of numerical and structural CA.

6) Sometimes the number of centrosomes per cell estimated appears extremely high (8=16 centrioles), leaving the possibility that DAB substrate aggregates may score as “centrosomes”, in particular as signal amplification and enzymatic detection has been routinely used. This needs to be excluded as a confounding factor. Background signals may be better estimated using paraffin embedded cells pretreated with PLK4 inhibitors, to deplete centrioles. On the other hand, rather than having PLK4 overexpression to amplify centrioles in an uncontrolled manner, induction of cytokinesis defects would allow linear scaling of centrosome numbers and may be useful to create internal standards for training.

- To address Reviewer 1#’s concern, we sought expert advice from Professor Susana Godinho (Queen Mary University of London), a leading expert in centrosome biology. While established protocols for generating paraffin-embedded cells pre-treated with PLK4 inhibitors are not routinely available, Professor Godinho provided guidance on the use of the selective PLK4

inhibitor centrinone (Wong *et al.* 2015 PMID: 25931445), to deplete centrioles in cell-based systems. Using this approach, we performed additional functional experiments in MCF10A and MCF10A-PLK4 cells. These new experiments and data, now included in the revised manuscript (new **Supplementary Fig. 5**; Results, lines 273-302; Methods, lines 808-809), provide orthogonal validation of centriole depletion and centrosome quantification following PLK4 inhibition (see more details in our response to Reviewer #3, Points 1 & 2). Importantly, this serves as a robust negative control demonstrating that CenSegNet does not erroneously classify diffuse or residual signal as centrosomes under conditions where centrioles are experimentally depleted.

To further exclude the possibility that DAB substrate aggregation is being misidentified as centrosomes, we included negative control FFPE samples processed by the Research Histology Facility using standard IHC workflows. These controls exhibited minimal background staining and no discrete centrosome-like puncta (revised **Supplementary Fig. 18a**). In addition, as highlighted in the revised Methods (lines 878-898), staining patterns and signal specificity in both control and experimental samples were independently reviewed by experienced pathologists, including Dr Constantinos Savva (Medical Oncology, University Hospital Southampton) and Dr Margaret Ashton-Key (Consultant Haematopathologist, University Hospital Southampton). Both confirmed that the quantified signals are fully consistent with bona fide centrosome staining rather than artefactual DAB deposition. Together, these additional histological controls, independent expert evaluations, and functional perturbation experiments, combined with our robust image annotation and modality-specific calibration strategies further highlighted in the revised Methods (lines 888-908; lines 948-969)—as also discussed in our responses to Reviewer #1, point 3 and to Reviewer #3, points 5, 6, 8—provide strong support that the signals detected by CenSegNet represent genuine centrosomes rather than DAB artefacts.

7) In summary, the CenSegNet seems to be a valuable tool for quantifying centrosomes in tissues. However, since structural aberrations are impossible to see with the microscopy techniques used here, we doubt the interpretation of the respective data (Figs 3-5). The use of antibodies recognizing actual centriolar proteins, such as CP110 or alike, are recommended to be included in addition to or to replace pericentrin.

- We thank Reviewer #1 for this constructive summary and for highlighting an important interpretative limitation, which we now explicitly address and contextualise. We fully agree that centriole-specific markers are required for direct assessment of centriole number and ultrastructure, and we recognise that routine-resolution chromogenic IHC cannot resolve centriole-level architectural features. As discussed above, this limitation is now explicitly acknowledged in the revised manuscript (Discussion, lines 547–563).

We wish to emphasise that centriole-specific validation was already incorporated into the initial submission. Specifically, we performed IF experiments using GT335, a well-established centriole marker, in MCF10A cells and in mouse mammary tissue (**Supplementary Fig. 1**, now revised to include lightning-based super-resolution imaging of GT335 and pericentrin). These experiments demonstrated the expected colocalisation between GT335-labelled centrioles and pericentrin-labelled PCM, providing orthogonal validation of centrosome identity and supporting the biological relevance of PCM-based phenotyping. However, despite extensive optimisation in close collaboration with the Research Histology Facility (University Hospital Southampton) and Professor Susana Godinho, GT335 staining in FFPE tissue sections did not yield sufficiently robust or reproducible signal to support reliable centriole detection or quantification at the tissue level in the TMA cores. We consistently observed variable signal intensity and elevated background, consistent with well-recognised technical challenges associated with detecting centriolar epitopes in FFPE material, where fixation-induced antigen retrieval and epitope degradation frequently impair antibody performance. Thus, while centriole-specific markers perform reliably in low-throughput tissue IF (Wang *et al.* 2019 PMID:

30699045; Denu *et al.* 2019 PMID: 29330283), their application in FFPE chromogenic IHC remains technically limited and is not currently standardised for tissue-level quantitative analysis.

Given these constraints, our tissue-based analyses necessarily rely on pericentrin, a widely used and technically robust PCM marker for centrosome identification in paraffin-embedded human epithelia. Accordingly, our interpretation focuses on centrosome number and PCM-defined size, which remain reliably detectable and quantifiable under FFPE conditions and are compatible with automated, high-throughput analysis in complex epithelial tissues. Importantly, and as discussed in the Introduction of the initial submission (revised lines 74-89), most existing centrosome-quantification tools are tailored to IF imaging and are not compatible with standard chromogenic IHC workflows used in clinical pathology, limiting their translational applicability. Our approach is therefore intentionally aligned with widely adopted IHC pipelines to address this gap.

To strengthen these tissue-based analyses, we complemented them with higher-resolution IF imaging datasets (revised **Supplementary Fig. 1**; new **Supplementary Fig. 5**), in which both centriole-specific (GT335) and PCM-specific (pericentrin) markers can be simultaneously visualised and quantitatively compared. Across multiple mammary epithelial models, including MCF10A-PLK4 and MCF10DCIS cells, these orthogonal datasets consistently confirm the expected relationship between centriole number and PCM organisation, thereby supporting the validity of our centrosome phenotyping framework.

Together, these technical considerations and complementary imaging data clarify why centriole-specific antibodies cannot currently replace pericentrin in FFPE tissue analysis, while demonstrating that our combined IHC and IF strategy provides a robust and biologically grounded framework for quantifying centrosome number and PCM enlargement. At the same time, we now state explicitly in the revised Discussion (lines 556-560) that direct assessment of centriole ultrastructure lies beyond the resolution limits of the present FFPE tissue-based methodology and will require future studies using electron, super-resolution, or expansion microscopy.

**Reviewer #3 (Remarks to the Author):**

1) Include functional assays (e.g., perturbation of PLK4, AURKA, or PCM components) to validate the inferred mechanistic uncoupling of numerical and structural CA.

2) Alternatively, clarify more explicitly in the Discussion that conclusions are observational and hypothesis-generating.

- To address <u>Reviewer #3's</u> points, we have now incorporated a functional PLK4 perturbation assay using the selective PLK4 inhibitor centrinone (Wong *et al.* 2015 PMID: 25931445) combined with immunofluorescence (IF) confocal imaging, allowing us to directly test the relationship between centriole number, centrosome number, and centrosome size. In this experiment, centriole amplification was first induced in Tet-On MCF10A-PLK4 cells using doxycycline. Both MCF10A and MCF10A-PLK4 cells co-labelled for pericentrin (PCM) and GT335 (centriole) were then treated with centrinone for five days, enabling targeted inhibition of PLK4 activity following amplification and thus functional perturbation of centriole biogenesis (new **Supplementary Fig. 5a**). Under these conditions, centrinone treatment did not significantly alter the number of centrosomes—defined by pericentrin—per cell in either cell line (new **Supplementary Fig. 5b**). In contrast, centrinone treatment led to a marked reduction in centriole number per centrosome (new **Supplementary Fig. 5c**), confirming effective inhibition of PLK4-dependent centriole duplication. Interestingly, PLK4 inhibition also resulted in a significant reduction in centrosome area in both MCF10A and MCF10A-PLK4 cells (new **Supplementary Fig. 5d**), demonstrating that centrosome size is sensitive to PLK4 activity and centriole content. To further examine the relationships between these parameters, we

performed correlation analyses. In control/DMSO-treated cells, centrosome area exhibited a strong positive correlation with centriole number per centrosome (MCF10A: $R^2 = 0.4442$; MCF10A-PLK4: $R^2 = 0.7752$; new **Supplementary Fig. 5e, f, i, g**), consistent with coordinated scaling between centriole content and PCM expansion. Upon centrinone-mediated PLK4 inhibition, however, this coupling was substantially weakened (MCF10A: $R^2 = 0.1941$; MCF10A-PLK4: $R^2 = 0.1825$; new **Supplementary Fig. 5g, h, k, l**). Similarly, while centrosome number per cell correlated positively with total centriole number in control conditions (MCF10A: $R^2 = 0.3528$; MCF10A-PLK4: $R^2 = 0.3544$; new **Supplementary Fig. 5e, f, m, n**), this relationship was significantly lost following centrinone treatment (MCF10A: $R^2 = 0.0405$; MCF10A-PLK4: $R^2 = 0.0952$; new **Supplementary Fig. 5g, h, o, p**). Collectively, these functional perturbation experiments provide evidence that PLK4 inhibition disrupts the quantitative relationships between centriole number, centrosome number, and centrosome size, while under unperturbed conditions centriole content and centrosome size are tightly coupled. We believe these data address the Reviewers' concern and support our observation that numerical and structural CA can be mechanistically uncoupled in non-physiological/perturbed contexts such us breast cancer. The revised manuscript has been updated accordingly to incorporate these new experiments (Results, lines 273–302; Discussion, lines 586-593; Methods, lines 808-809).

3) Provide quantitative CA analyses or clinical correlations for at least one additional epithelial cancer type.

4) Alternatively, reframe claims of generalisability more conservatively.

- We thank Reviewer #3 for this helpful suggestion and have now included additional evidence to address this point, which also complements our response to Reviewer #1 (point 5), where we provide the first, CenSegNet-based quantitative analyses correlating numerical and structural CA with clinical outcomes in our breast cancer cohort. While our study provides primary, cohort-level clinical correlations in breast cancer, quantitative analyses of CA and its clinical relevance have been reported across multiple epithelial malignancies. For example, as discussed in the introduction of the initial manuscript, Mittal *et al.* developed a quantitative centrosome amplification score (CAS) using IF confocal microscopy combined with digital image analysis and demonstrated that numerical and structural CA can be robustly measured and linked to clinically relevant tumour features across solid cancers (Mittal *et al.* 2021 PMID: 33106971). In colorectal cancer, centrosome abnormalities have been quantitatively assessed and shown to correlate tightly with chromosomal instability and aneuploidy. CA and instability occur preferentially in aneuploid colorectal tumours and cell lines and are associated with numerical chromosomal aberrations and tumour progression (Ghadimi *et al.* 2000 PMID: 10612807). Additionally, common genetic alterations in colorectal cancer (e.g. APC and β-catenin mutations) disrupt centrosome regulation, leading to centrosome over-duplication and accumulation (Harrison *et al.* 2017 PMID: 29128368). In prostate cancer, centrosome defects have been identified in early, pre-invasive epithelial lesions and shown to co-occur with mitotic spindle defects, cytological abnormalities, and chromosomal instability, supporting their role in tumour initiation and progression (Pihan *et al.* PMID: 12649205). Similarly, in ovarian cancer, centrosome defects have been quantitatively documented in patient samples, where they associate with higher tumour grade, chromosomal instability, and disease progression (Hsu *et al.* 2005 PMID: 15499629).

Collectively, these studies demonstrate that CA is a quantitatively measurable and clinically relevant trait across diverse epithelial cancers, supporting the broader relevance of high-throughput centrosome quantification approaches. At the same time, we recognise that direct generalisation of CenSegNet across tumour types requires careful consideration of tissue-specific context, marker performance, and clinical annotation. Accordingly, we have revised the manuscript to frame claims of generalisability more conservatively, explicitly stating that while accumulating evidence supports the applicability of quantitative CA analysis across

epithelial malignancies, further validation in large, cancer-specific cohorts will be required to establish CenSegNet's predictive or prognostic applicability beyond breast cancer (Discussion, lines 723–735).

5) Include inter-observer agreement metrics (e.g., IoU, Cohen's κ) on a subset of data.

6) Clarify whether annotations were reviewed or validated by multiple experts.

- The initial annotations were performed by Jiaoqi Cheng and subsequently reviewed by two senior domain experts: Dr Constantinos Savva (SCE, Medical Oncology) and Dr Margaret Ashton-Key (a nationally and internationally recognised consultant Haematopathologist), both of whom have extensive clinical experience. In addition, the annotations were further validated by two collaborators with complementary expertise in cell biology (Mengyang Gou, Annotator 1) and computational analysis (Peixin Zuo, Annotator 2). To ensure annotation quality and consistency, any disagreements were carefully examined and resolved through discussion with the senior experts. These details have now been incorporated into the revised manuscript to enhance transparency and reproducibility (Methods, lines 888-908; Results, lines 144-152).

To further quantify annotation reliability in IHC and IF images, we have included a formal analysis of inter- and intra-observer agreement based on independent annotations from Annotator 1 and Annotator 2. Both annotators independently annotated the same subset of images, and their annotations were compared with those of the main reference annotator (new **Supplementary Fig. 2**). Each annotator also performed a repeat round of annotation to assess intra-observer consistency. Agreement was quantified using the F1 score, IoU, and Cohen's k at the patch level (256 × 256 pixels). This validation demonstrated high intra- and inter-observer consistency across datasets. In the immunohistochemistry datasets, mean F1 scores and Cohen's κ were close to or exceeded 0.90, with a mean IoU of approximately 0.70 (new **Supplementary Fig. 2a, b**). In the immunofluorescence datasets, mean F1 scores and Cohen's κ were close to or exceeded 0.95, with a mean IoU of approximately 0.92 (new **Supplementary Fig. 2c, d**). For epithelial (haematoxylin-stained) segmentation, mean F1 scores and Cohen's κ were close to or exceeded 0.91, with a mean IoU of approximately 0.87 (new **Supplementary Fig. 2e, f**). Repeat annotations by the same annotators yielded comparable results, indicating strong intra-observer reproducibility. Together, these analyses demonstrate that the annotation approach is robust and reproducible across different annotators and supports the reliability of the dataset used for CenSegNet development and evaluation. This has now been clarified in revised manuscript (Results, lines 144-152; Discussion, lines 547-553). The revised Methods (lines 888-908) also explicitly states that annotations were performed independently by multiple annotators, evaluated against a reference annotator, and repeated to assess consistency.

7) Provide sensitivity analyses showing how varying this threshold affects conclusions.

- We thank Reviewer #3 for this helpful suggestion. We have now added more comprehensive sensitivity analyses, to complement those included in the initial submission's **Fig. 2.**, to assess whether the study conclusions depend on the choice of detection or segmentation threshold. Specifically, we evaluated four representative image types: immunohistochemistry, confocal immunofluorescence, lightning-based super-resolution immunofluorescence, and haematoxylin-stained images. For each experiment, we varied either the detection threshold or the segmentation threshold from 0.1 to 0.8 while keeping the other threshold fixed at 0.15 (default value), and assessed performance using F1 score, IoU, and Cohen's κ (new **Fig. 3**). Our sensitivity analyses demonstrate CenSegNet's robust performance over varying threshold values across all four image types. In immunohistochemistry images, performance remained stable as the detection threshold increased from 0.1 to 0.5, with reductions observed only at higher thresholds (new **Fig. 3a, b**). When the segmentation threshold was varied, F1 score, IoU, and Cohen's κ remained stable across the tested range (new **Fig. 3a, c**). A similar pattern was observed for both confocal (new **Fig. 3d-f**) and lightning-based super-resolution (new **Fig.**

**3g-i**) immunofluorescence images. F1 score and Cohen's κ remained stable across detection thresholds from 0.1 to 0.8, with only minor fluctuations in IoU (new **Fig. 3d, e, g, h**), while variation of the segmentation threshold over the same range had little effect on performance (new **Fig. 3d, f, g, i**), indicating that segmentation accuracy was not sensitive to threshold selection. For epithelial segmentation in haematoxylin-stained images, performance was stable across detection thresholds from 0.1 to 0.3 and decreased at higher values (new **Fig. 3j, k**). Similarly, segmentation performance remained stable when the segmentation threshold was varied from 0.1 to 0.4, with decreases observed only at larger values (new **Fig. 3j, l**). Together, these analyses demonstrate that our main conclusions are not dependent on a single threshold choice. The selected threshold of 0.15 lies within the stable performance range for all tested image types and metrics. We have revised the text to incorporate these analyses (Results, lines 224-246; Discussion, line 549; Methods, lines 998-1006)

8) Justify modality-specific calibration or confirm robustness across IF and IHC datasets.

- In this study, we explicitly accounted for the fundamental differences between IF and IHC imaging modalities through a modality-specific calibration strategy, rather than applying a single uniform pipeline. IF and IHC differ substantially in signal generation, intensity distributions, and noise characteristics. IF images rely on fluorescence emission and typically exhibit channel-dependent variability, a broader dynamic range, and sensitivity to photophysical effects. In contrast, IHC images are based on chromogenic staining, resulting in distinct colorimetric contrast, comparatively lower dynamic range, and different background profiles. Applying a single calibration strategy across both modalities would therefore introduce systematic bias and compromise model performance. To address this, we implemented modality-specific preprocessing pipelines, tailored to each imaging type prior to model training. For IHC images, preprocessing steps included contrast enhancement and background suppression to improve signal discrimination. Images were partitioned into overlapping 256 × 256-pixel patches, and regions containing low-quality signals or technical artefacts were excluded to ensure dataset integrity. In addition, data augmentation strategies (e.g. HSV colour jitter and geometric transformations) were applied to improve robustness to staining variability and imaging heterogeneity. For IF images, preprocessing was designed to account for fluorescence-specific signal properties while preserving biologically relevant structural information. IF images were similarly cropped into overlapping patches, and RGB channels were converted to grayscale to reduce inter-channel redundancy and improve computational efficiency. This step also reduces sensitivity to channel-specific intensity fluctuations, while retaining the spatial features required for centrosome detection. Together, these modality-specific calibration steps ensure that the model receives appropriately normalised inputs for each imaging modality, thereby improving generalisability and minimising modality-driven bias. We have clarified further this rationale and the corresponding preprocessing steps in the revised manuscript (Methods, lines 939-969; Results, lines 153-156).

9) Include multivariate analyses (e.g., Cox regression or logistic regression) controlling for standard prognostic factors.

- As suggested by Reviewer #3, we performed multivariable Cox proportional hazards regression analyses incorporating clinically relevant prognostic factors to evaluate the association between CA status in the tumour core and edge regions and clinical outcomes (new **Supplementary Fig. 13**). Our observations show that neither numerical CA nor structural CA was significantly associated with recurrence-free survival (RFS) or overall survival (OS) after adjustment for established clinicopathological variables, including age, prior breast cancer, smoking status, genetic status, tumour grade, tumour size, T stage, and nodal status. Notably, several well-established prognostic factors also did not reach statistical significance (Karihtala *et al.* 2020 PMID: 33046470; Bryan *et al.* 1986 PMID: 3008902), which likely reflects the limited

cohort size and number of events (see revised Methods, lines 1059–1071). Given these constraints, inclusion of multiple covariates reduced model stability, as evidenced by convergence challenges and widened confidence intervals. To maximise interpretability, we therefore conducted additional exploratory stratification analyses examining CA patterns across tumour core and edge regions. Specifically, we compared RFS and OS across combinations of numerical and structural CA states within these spatial compartments. These analyses have been incorporated and discussed in the revised manuscript (Results, lines 413–437; Discussion, lines 704-735; see also response to Reviewer #1, point 5). In these stratified analyses, lower structural CA was significantly associated with more favourable overall survival (new **Supplementary Fig. 12f**). In addition, lower numerical CA combined with higher structural CA was associated with comparatively poorer survival patterns and adverse clinicopathological features, including larger tumour size and increased prevalence of family history (see **Fig. 5i, j, m, o**), although not all comparisons reached statistical significance. Taken together, while these findings should be interpreted cautiously due to limited statistical power, the identification of a statistically significant association between structural CA and OS, together with coherent spatial and clinicopathological trends, suggests that spatially resolved CA patterns—particularly structural CA—may capture biologically meaningful aspects of tumour behaviour. Accordingly, these results warrant further investigation in larger, well-powered cohorts for more robust multivariate modelling (e.g. Cox or logistic regression) and assessment of the independent prognostic value of numerical and structural CA.

10) Clarify whether CA provides independent predictive value.

- In our BeGIN breast cancer cohort, CenSegNet-based analyses indicate that numerical and structural CA correlate with multiple clinicopathological variables rather than acting as isolated predictors. Notably, while lower levels of structural CA were significantly associated with more favourable overall survival, CA phenotypes remained closely linked to age, tumour size, nodal status, hormone receptor status, and genetic background. Age showed a distinct and potentially stage-specific association with CA phenotypes: numerical CA incidence peaked between 50 and 70 years of age (see **Fig. 4**), coinciding with the menopausal transition, while centrosome size in this age group was relatively smaller than in other age ranges. These observations suggest that age-associated physiological factors, such as hormonal changes or altered cellular homeostasis, may influence centrosome regulation and CA phenotypes.

Beyond our cohort, large-scale studies support the clinical relevance of CA across epithelial cancers. A pan-cancer TCGA analysis of 9,721 tumours across 32 cancer types developed a centrosome amplification–associated transcriptional signature (CA20) as a quantitative surrogate for CA, demonstrating significantly elevated CA scores in tumours compared with matched normal tissues and strong associations with genomic instability and tumour aggressiveness (de Almeida *et al.* 2019 PMID: 30856170). This study provides the first large-scale quantitative evidence linking CA to malignant phenotypes across epithelial cancers.

Direct clinicopathological correlations have also been established in specific tumour types. In bladder carcinoma, centrosome amplification and variability are significantly enriched in high-grade tumours and are strongly associated with chromosomal instability (Kawamura *et al.* 2003 PMID: 12705995). Earlier studies across epithelial malignancies reported that centrosome abnormalities correlate with tumour grade, loss of tissue differentiation, and chromosomal instability (Lingle *et al.* 2002 PMID: 11830638; Lingle & Salisbury 1999 PMID: 10595924), and that CA is more pronounced in advanced and recurrent tumours and associated with aggressive malignant behaviour in xenograft models (Chan *et al.* 2011 PMID: 22043171).

Importantly, functional studies indicate that CA can act as a biological driver, rather than a passive correlate, of tumour progression. In mammary epithelial systems, CA was shown to be sufficient to induce invasive behaviour via increased centrosomal microtubule nucleation and Rac1 activation (Godinho *et al.* 2014 PMID: 24739973). Adaptive mechanisms that allow

tumour cells to tolerate CA—such as E-cadherin-loss-dependent centrosome clustering—have also been described (Rhys *et al.* 2018 PMID: 29133484), with more recent work linking CA to tumour–stroma signalling through extracellular vesicle secretion (Adams *et al.* 2021 PMID: 33592190) and to enhanced nuclear migration in confined environments (Monteiro *et al.* 2023 PMID: 37403793).

Consistent with this biology, two mechanistically grounded therapeutic strategies have emerged: suppression of centriole biogenesis, most directly via inhibition of the master regulator PLK4, and pharmacological disruption of centrosome clustering. Selective PLK4 inhibition using centrinone induces progressive centriole loss and mitotic failure (Wong *et al.* 2015 PMID: 25931445), while translational advances with the oral PLK4 inhibitor CFI-400945 demonstrated in vivo antitumour activity and favourable pharmacokinetics (Mason *et al.* 2014 PMID: 25043604), with early-phase clinical trials showing tolerability and preliminary efficacy in advanced solid tumours (Veitch *et al.* 2019 PMID: 31303643) and haematologic malignancies (PMID: 38114624). Complementary approaches targeting centrosome clustering, including inhibition of the kinesin-14 motor KIFC1/HSET, induce lethal multipolar mitoses selectively in CA-high tumour cells (Rebacz *et al.* 2007 PMID: 17616693; Wu *et al.* 2013 PMID: 23895133; Watts *et al.* 2013 PMID: 24210220). These therapeutic aspects of CA have been highlighted in the initial submission (now revised Discussion, lines 748-752).

Taken together, these quantitative pan-cancer analyses, epithelial cancer–specific clinical correlations, and mechanistic studies support CA as a robust marker of tumour aggressiveness and therapeutic vulnerability. However, given the close interplay between CA and established clinicopathological variables, we now further clarify in the revised manuscript that evidence supports CA as a complementary and biologically informative feature, rather than a definitively independent predictive marker (Discussion, lines 717-719). We further state that larger, clinically well-annotated cohorts will be required to determine whether Numerical CA or Structural CA provides independent prognostic or predictive value beyond standard clinical factors (Discussion, lines 720-723; lines 751-755).

11) Clarify inference-time requirements for typical users.

- To address Reviewer #3's point, we have now explicitly described the hardware requirements and runtime characteristics relevant to typical usage in the revised manuscript (Methods, lines 1007-1016; Results, lines 475-505). By "typical users" of CenSegNet, we refer to biology scientists, biomedical researchers, clinical researchers, pathologists, and clinicians who use IHC or IF images in research, translational, or diagnostic-support workflows.

To provide practical guidance for these users, we benchmarked CenSegNet on several representative computing environments with different levels of accessibility and performance. First, to represent a standard local computing environment commonly available to biology scientists and biomedical researchers, we tested CenSegNet on a routine office laptop purchased in 2021 and used for daily work. This laptop was equipped with an 11th-generation Intel® Core™ i5-1145G7 processor and 8 GB RAM. Second, because some university-based researchers may have access to institutional CPU servers, we evaluated CenSegNet on a higher-performance CPU server equipped with an AMD EPYC™ 9334 32-core processor and 128 GB RAM. Third, to represent a freely accessible GPU-based option for users without dedicated local GPU hardware, we tested CenSegNet using an NVIDIA Tesla T4 GPU with approximately 16 GB VRAM, corresponding to the standard free-tier GPU provided by Google Colab. The benchmarking results are reported in new **Supplementary Fig. 16**. These additions provide clear and practical guidance for users seeking to deploy CenSegNet in real-world research or clinical-adjacent environments.

These inference-time expectations are further quantified through explicit CPU-only and lower-end GPU benchmarking, as detailed in response to Reviewer #2 point 12 below.

12) Provide benchmarks on lower-end GPUs or CPU-only inference where possible.

- In our original experiments, CenSegNet was executed on a system equipped with four NVIDIA A100 GPUs, each with 40 GB VRAM, achieving satisfied inference time per sample across the IHC (0.28-0.82 min per sample), IF (0.19-0.26 min per sample), and epithelial (haematoxylin-stained; 0.24-0.99 min per sample) datasets. The corresponding input image resolutions were 3370 × 4487 to 6630 × 5941 pixels for the IHC and epithelial datasets, and 1024 × 1024 pixels for the immunofluorescence dataset. We agree that additional benchmarking on more commonly accessible hardware is essential for assessing the practical usability of the method. To address this point, we have now included explicit inference-time benchmarks on three representative configurations, comprising two CPU-only settings and one lower-end GPU setting. These include a standard office laptop, a higher-performance CPU server, and a freely accessible NVIDIA Tesla T4 GPU available through Google Colab.

These analyses are shown in new **Supplementary Fig. 16**. Specifically, we evaluated inference performance on three representative platforms: (i) CPU #1, a standard consumer-grade office laptop purchased in 2021 and used for routine daily work, equipped with an 11th-generation Intel® Core™ i5-1145G7 processor and 8 GB RAM; (ii) CPU #2, a higher-performance server system equipped with an AMD EPYC™ 9334 32-core processor and 128 GB RAM; and (iii) GPU, an NVIDIA Tesla T4 with approximately 16 GB VRAM, which is the standard free-tier GPU commonly available through Google Colab. CPU #1 was intentionally included to reflect a modest, readily available laptop-class device rather than optimized workstation hardware. As expected for such a lower-end local configuration acquired around five years ago. Inference on this configuration was fully feasible, requiring approximately 2.6–8.8 min per sample for the immunohistochemistry dataset, 2.1–3.5 min per sample for the immunofluorescence dataset, and 5.8–14.0 min per sample for the epithelial dataset (new **Supplementary Fig. 16a**). The corresponding input image resolutions were 3370 × 4487 to 6630 × 5941 pixels for the immunohistochemistry and epithelial datasets, and 1024 × 1024 pixels for the immunofluorescence dataset. On the higher-performance CPU server, runtime was substantially reduced to approximately 1.2–3.3 minutes per sample for the immunohistochemistry dataset and 0.9–1.2 minutes per sample for the immunofluorescence dataset, and 2.0–5.5 min per sample for the Epithelial dataset (new **Supplementary Fig. 16b**). Notably, inference on the freely accessible Tesla T4 GPU provided by Google Colab achieved sub-minute processing per sample for both datasets (new **Supplementary Fig. 16c**).

Together, these results indicate that CenSegNet remains practically deployable on typical user hardware, including older or modest laptop CPUs. Importantly, practical deployment does not require dedicated institutional GPU hardware or other highly specialised computing resources, as substantial acceleration can also be achieved using freely available cloud GPU resources such as Google Colab. We have revised the manuscript accordingly to explicitly clarify these inference-time characteristics and to highlight the deployability of CenSegNet in standard CPU-only environments as well as accessible cloud-based GPU settings (Methods, lines 1007-1016; Results, lines 475-505).
